# Gender differences in access to extension services and major farm inputs among paddy seed growers in Rautahat district of Nepal

**DOI:** 10.3389/fsoc.2026.1705290

**Published:** 2026-03-18

**Authors:** Sandeep Timalsina, Rajesh Paudel, Hari Krishna Panta, Milan Subedi, Jay Chaurasia

**Affiliations:** 1 Department of Agricultural Extension and Rural Sociology, Institute of Agriculture and Animal Sciences, Tribhuvan University, Kathmandu, Nepal; 2 Department of Agricultural Economics, Institute of Agriculture and Animal Sciences, Tribhuvan University, Kathmandu, Nepal

**Keywords:** extension, farm inputs, gender, paddy, seed

## Abstract

**Introduction:**

Extension services for paddy seed farmers are essential for boosting agricultural productivity; however, significant gender gaps in access to these services exist in Nepal. This study, carried out in 2024 in Rautahat district, explored the role of gender and socio-economic characteristics in determining access to paddy seed extension services and major agricultural inputs.

**Methods:**

A convergent parallel mixed-methods research design was used. Two hundred and sixty-five farmers were surveyed with a structured questionnaire using a multistage sampling method. Qualitative data were gathered through focus group discussions and key informant interviews. Data analysis included descriptive statistics and binary logistic regression.

**Results:**

The results showed that gender, income, age, ethnicity, and farm type influenced access to extension services, with the logistic model explaining 41.6% of the variation. Although men had higher access to these services, women were more likely to utilize them once accessed. Education, farm size, and off-farm employment had limited influence on perceived access. The farm inputs access model accounted for 42.0% of the variation, with participation level, farm type, and ethnicity being the strongest predictors.

**Discussion:**

Perception analysis revealed that women held more positive attitudes toward extension services but faced structural and social barriers, such as fewer female extension agents, financial constraints, and limited decision-making power, which hindered their ability to make data-driven decisions.

**Conclusion:**

The findings highlight the need for gender-sensitive and appropriate extension strategies to ensure equitable access for all farmers, ultimately improving access and productivity, especially for women and marginalized groups.

## 1 Introduction

Sex denotes the physical differentiation of individuals as male and female, while gender pertains to identities, roles, and opportunities, which are socially and culturally constructed and exist on a spectrum of non-binary (Shrestha and Gartoulla, 2015). These gendered aspects are deeply embedded within the socio-cultural context and dramatically shape the livelihoods, healthcare, and resource accessibility. To illustrate, women face the dual burden of being more likely to experience widowhood, chronic illness, and the burden of caregiving, but also demonstrate psychological and physical resilience through self-protective processes and alternative well-being strategies (Gezimu et al., 2021). In agriculture, the contributions of women are considerable; at the global level, women participate more frequently than men in farming and also serve in important capacities in agribusiness as well as in the processing and marketing sectors and as supporters in the family network (Odame et al., 2002). Although women have played and continue to play a pivotal role, they have almost universally been excluded from agricultural extension services, which hampers their access to training, credit, and the ability to participate in decisions (Cohen and Lemma, 2011).

The importance of disseminating agricultural knowledge and advanced technology is challenging, especially in the face of adopting improved agricultural practices. Extension Services help in the customization of information that will directly aid in the improvement of production efficiency, household income, and the welfare of people in rural areas (Rahman et al., 2020). In Nepal, rice (paddy) is the staple crop that supports a significant portion of agricultural GDP and contributes to food security. Extension services help in the dissemination of quality seeds and promotion of better management practices (Timalsina and Shrestha, 2023). Within Nepal, the Prime Minister Agriculture Modernization Project (PMAMP) has marked rice production enhancement and has designated Rautahat district as a zone for the proliferative production of paddy seeds in the fiscal year 2019/20 A.D (Timalsina and Shrestha, 2023). Despite such critical interventions, the role of extension still faces the challenge of nearly 75% of farmers in the country being unreached, which is a significant hindrance to agricultural progress (Babu and Sah, 2019).

After the federal restructuring of Nepal in 2015, the 2017 Act on Local Governance also decentralized agricultural extension services to local governments in the bid to achieve better democratization, accountability, and accessibility (Acharya and Zafarullah, 2020; Jaishi et al., 2015). However, those extensions remained gender unequal. Women farmers in the Terai region, particularly Rautahat, face a unique set of challenges to accessing agricultural extension services because of deep-rooted cultural norms, limited mobility, and a workforce of predominantly male extension staff (Dhungana, 2022; Thapa, S. et al., 2020). Research demonstrates that these women, and more generally women-headed households, face a striking inequality in the number of extension visits compared to their male counterparts, along with a lack of access to basic farming inputs of a certain standard (Paudyal et al., 2019). Such barriers curtail women’s involvement in the seed value chains and the adoption of better farming practices, which deepens the disparity in productivity and income (Thapa, S. et al., 2020).

International assessments support these conclusions. The FAO reported in 2019 that women face systematic exclusion from training and other resources relative to men. In Nepal, limited service centers, male-dominated extension staffing, program timings, and the narrow scope of the content presented are additional barriers to women’s inclusion (Ragasa et al., 2013). In addition, women’s reduced access to credit, high input costs, and limited autonomy over their financial or technological decisions face strong structural constraints (Devkota et al., 2018; Gurung, 2023). Women’s roles in agriculture are largely overlooked, including tasks such as transplanting, weeding, and harvesting, while men dominate land preparation, irrigation, and marketing roles (Thapa, S. et al., 2020; Tamang, 2019).

The major impacts of this gendered division of labor and continued exclusion from extension are not only reflected in the empowerment of women but also pose a barrier to agricultural productivity and food security in rural Nepal (Paudyal et al., 2019). Despite several studies that have identified prevailing gender disparities in agricultural services at the national and regional level, there is very little evidence available in relation to paddy seed growers in the Terai, especially those located in the Rautahat district. With women making up more than 80% of the agricultural labor force in Nepal, consideration of gendered access to extension services is essential for designing inclusive interventions (FAO, 2019). Thus, this study aims to examine how paddy seed producers in Rautahat district differ by gender in their access to agricultural extension services. In order to support inclusive agricultural development, increase productivity, and fortify household food security, this research attempts to identify the sociocultural, economic, and institutional barriers that women encounter. This study hypothesized that paddy seed-producing men and women farmers would have differences in access to extension services. The following research questions were made to obtain the research objectives of this study:

1. How do socio-economic factors affect gender disparities in access to paddy seed extension services?
2. What role does gender play in access to major farm inputs?

## 2 Literature review

### 2.1 Global and Nepalese perspectives on gender in agricultural extension

In Nepal, the agricultural feminization process has been trending up due to male out-migration, but women’s access to extension services and other services has been curtailed. Some evidence continues to suggest that female-headed households are less likely to use labour-saving technologies and mini-tillers without structured support (Paudel et al., 2020). Along with an overall decrease in extension information relayed to women during the COVID-19 pandemic, the access to information for women in Dang district severely declined, which disallowed women an opportunity for many potential productive tasks (Alvi et al., 2021). More comprehensive explanations of women as they change roles while emerging as managers in Nepal’s eastern hills and Jhapa district provide many emerging and engaged functions, which will invariably limit their empowerment in these changing roles due to an absence of valid advisory systems on women’s mobility and social networks (Upreti et al., 2018; Gartaula et al., 2012). In a multi-species system, where there are great inter-dependence factor between species not least in a complex matrix of links and relationships (given the lack of female extension agents), the chances for women’s engagement and opportunities for the transfer of knowledge will be limited and invariably undermine the findings which illustrate systemic gaps in women-specific structural model of service (Tiwari and Shingh, 2020).

International evidence indicates that delivery mechanisms inform and often determine the gender consequences of extension. Randomized trials in Ethiopia, and others, supply evidence that video-mediated approaches can scale up information provision and generate meaningful adoption gains for women farmers (Abate et al., 2023; Baul et al., 2024). Household-level interventions that targeted information provision to wives, or explicitly identified female agents, enhanced women’s participation and nutrition decisions (Ahmed et al., 2023; Lecoutere et al., 2023). Immaterial functional service, demand-led models of extension -like plant health clinics- increased uptake, benefits, productivity, or food security benefits. Nevertheless, in cases where there is at least a conscious gendered design, there could still be male-biased benefits (Tambo et al., 2021). At a similar level, the six diverse contextual examples really demonstrate the primary principles of inclusive targeting, enhancing women’s role in the extension workforce, and innovative delivery through ICT for age equitable gains for women and men.

### 2.2 Extension services among paddy seed farmers

The extension services to paddy seed farmers in Nepal are directed by landholding, gender, and organizational back-up. For example, from studies from the Terai districts, male farmers had a better chance of mobilization into farmer groups and training, while women undertook numerous household responsibilities and faced mobility issues (Dhakal and Mishra, 2022). The study also showed that farmers’ understanding of improved seed production practices was inconsistent and that many farmers had connections to informal networks and less to government extension work (Dar et al., 2020). The Agriculture Development Strategy (ADS) has recognized improvements in the seed sector, but the lack of female extension workers and the weak link between farmer and extension work have limited women’s involvement in research and development (Joshi et al., 2016). There is some evidence also to suggest that co-operative-based extension to enhance seed services could manage seed quality and productivity; however, these developments need to be carefully implemented to make sure women are equally considered (Gautam et al., 2025). All the studies appear to point to the need to further employ gender-sensitive approaches to maximize expensive extension services in paddy seed systems.

Outside Nepal, seed farmers are facing similar intersections of gender and institutional capacity. The evidence emerging from South Asia indicates that women participating in seed extension pathways can, if the advisory service meets women’s responsibilities relating to seed selection and post-harvest management, result in increased varietal adoption and more productive outcomes (Timsina et al., 2023). ICT, as in the case of video-based extension interventions, is giving farmers “access to timely seed-related knowledge” in Bangladesh and India (including women) (Baul et al., 2024). Swath assessments using gender dimensions indicate that, when both spouses engage together in the training, there is improved uptake of improved rice varieties and seed systems (Ahmed et al., 2023). Similarly, demand-led extension activities encourage improved uptake of seed and input systems even with evidence of structural gender gaps (Tambo et al., 2021). These examples of evidence from the evaluations reinforce the general advantages of gender responsive extension and the value of the applied advances in paddy seed systems internationally.

### 2.3 Theoretical review

#### 2.3.1 Social role theory (1987)

Social Role Theory, developed mainly by Alice Eagly and her colleagues, holds that behavior, attitudes, and roles are all dictated by societal norms and expectations based on gender, status, or other social categories. It is one of the most dominant theories of gender variation because it explains the creation and perpetuation of traditional gender roles based on socialization and structural constraints. For instance, findings from cross-cultural studies show that women are overrepresented in caregiving occupations (e.g., nursing, teaching) and underrepresented in leadership positions, which aligns with socialization expectations that women should be communal (nurturing, empathetic) and men agentic (assertive, competitive).

Many countries’ traditional gender roles ascribed to men the main responsibility of food production and decision-making, while women were relegated to domestic or supportive roles. These social expectations, grounded in the Social Role Theory, create structural barriers to women’s access to extension services even though women undertake much of the agricultural work, particularly in developing countries.

#### 2.3.2 Penchasky and Thomas’s theory of access

Researchers have employed the work of Penchansky and Thomas (1981), namely their ‘five As’ model of access: availability, accessibility, accommodation, affordability, and acceptability, for years. These are the most important dimensions for defining access to any public service, as suggested by Penchansky and Thomas. Access is a multifaceted concept, and hence it cannot be measured. However, employing the theory of Penchansky and Thomas, this study has been able to define access in terms of varying dimensions so that access can be measured.

Each component refers to access achievement in its different yet interrelated forms (Saurman, 2016). Such factors do not readily lend themselves to easy binary classification as to whether or not access has been gained; instead, they represent a continuum. Access will be determined on comparative grounds, with some individuals experiencing simply ‘enhanced or limited access’.

Applied to the gender dimension of access to extension services at the local level, this theory proposes that women’s access may not only be curtailed by physical distance, but also by social and economic barriers imposed by cultural norms and perception of the services offered. Understanding the concept of access in all its dimensions to ensure equal access to extension services for all genders in all communities is essential.

## 3 Materials and methods

### 3.1 Study area

**Figure 1:**
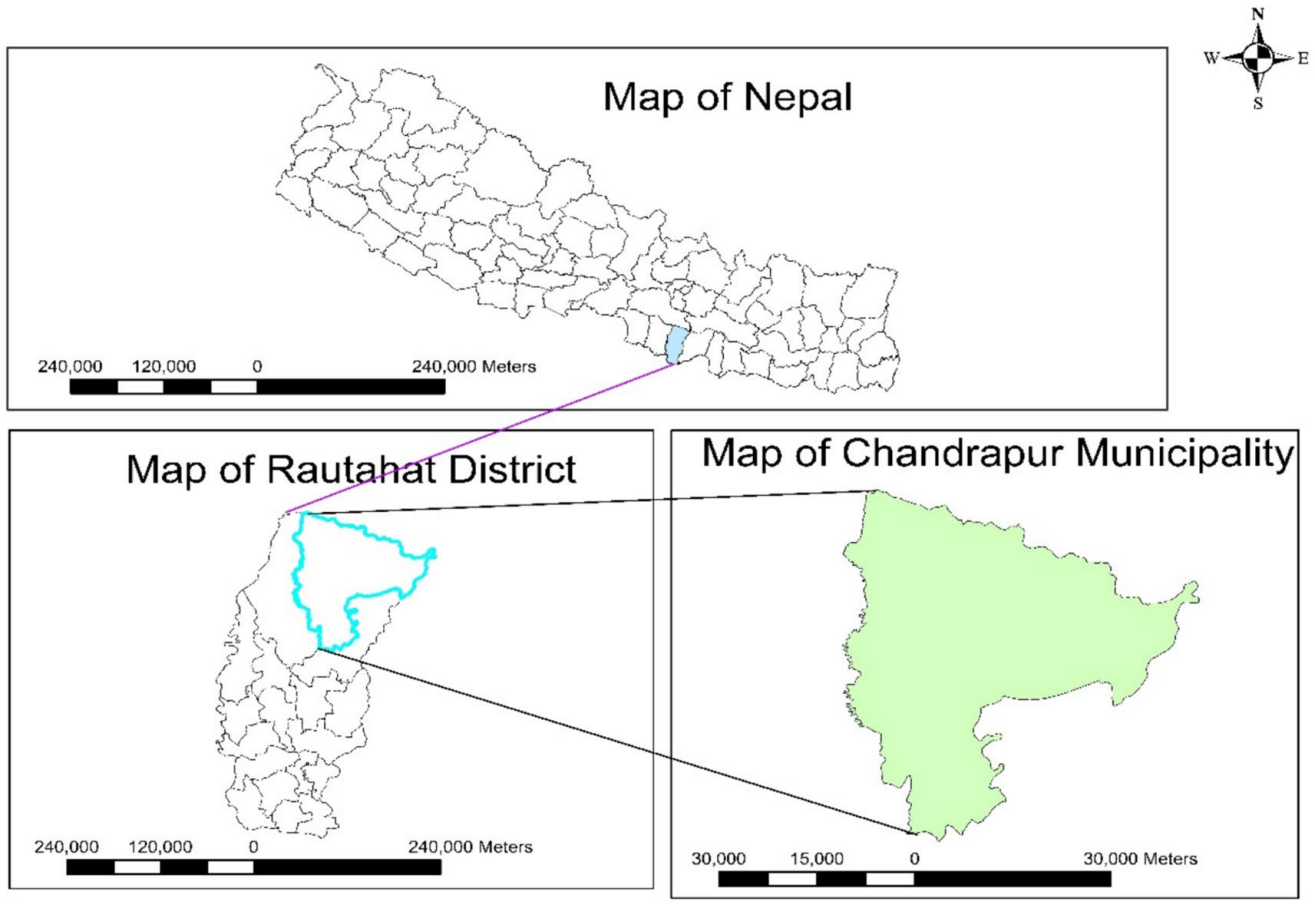
Location of the study site.

According to the Food and Agriculture Organization (FAO), Rautahat accounts for approximately 4.08% of the total harvested rice area in the country, ranking it 13th among Nepal’s rice-producing districts. There is a national project, PMAMP (Paddy seed Zone) at Garuda that provides extension programs to paddy seed farmers in different municipalities of it. Within Rautahat, Chandrapur Municipality was selected at random for the data collection. Chandrapur Municipality is located at 19.9615° N longitude and 79.2961° E latitude, which lies in the Madhesh Province. ArcGIS 10.4.1 software was used to prepare the map of the study site, which is shown in Figure 1.

### 3.2 Research philosophy, design, and approach

With an emphasis on the individual nature of reality and understanding, my study employed post-positivist and interpretivist philosophy. The development of post-positivist knowledge involved the observation and measurement of objective reality, with a focus on the significance of quantitative measurements and the examination of individual behavior (Creswell, 2014). A rigorous and systematic investigation of paddy-growing farmers’ access to extension and advisory services was conducted using empirical evidence to improve understanding of the phenomenon from a post-positivist perspective. Similarly, the interpretivist perspective aimed to develop an in-depth understanding of how farmers perceived access to extension and advisory services and to gain a comparative understanding of access as perceived by members and non-members.

**Figure 2:**
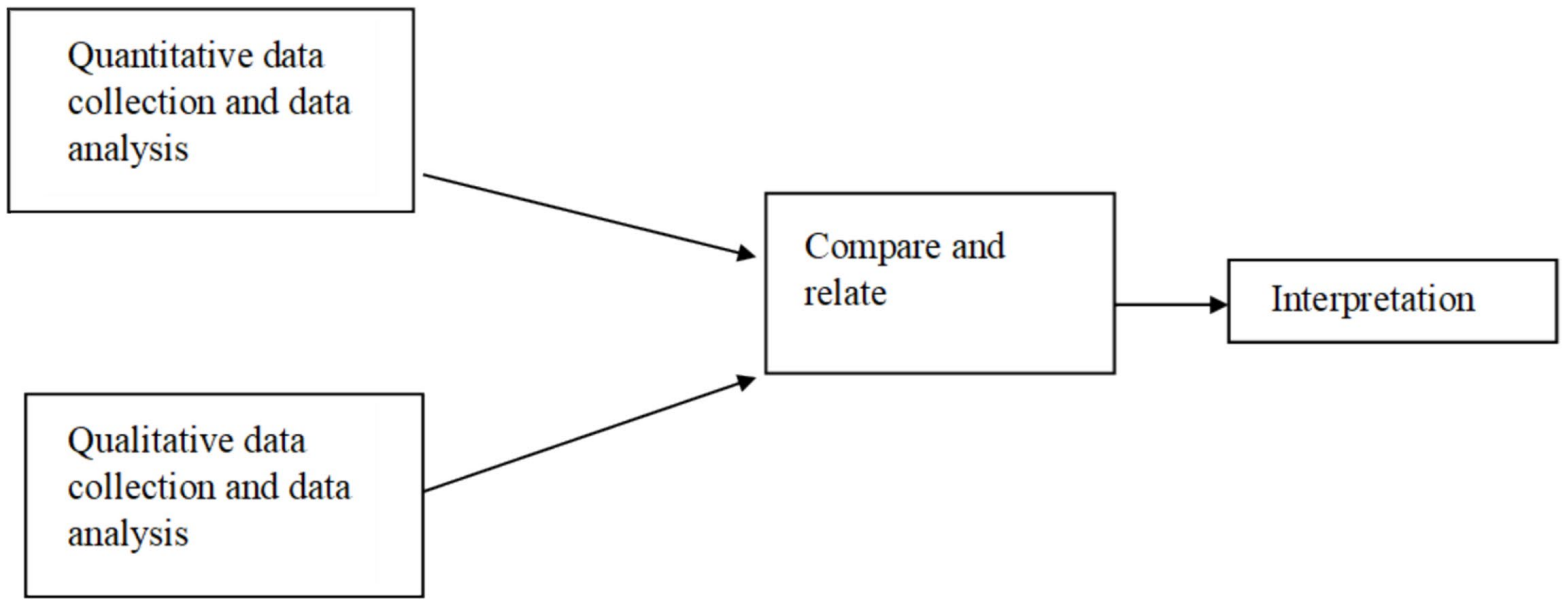
Convergent parallel mixed-methods research design.

The study used a descriptive, cross-sectional, non-experimental survey design. A convergent parallel mixed-method research design was used to collect the data from the respondents (Figure 2). Quantitative study in the analysis sought farmers’ access to extension and advisory services and perception towards access through a representative sample of farmers in the study area, while qualitative analysis aimed to provide an in-depth understanding of the subject under study. By incorporating both qualitative and quantitative research methods, this strategy broadens understanding by allowing one method to better understand and build on the results of the other.

Mixed methods research improves the overall research process by utilizing the strengths of different methods. Among the six mixed-method designs, a convergent parallel mixed-method design was used. Both qualitative and quantitative data are merged or integrated to compare results when using the convergent parallel mixed method. This design employs quantitative and qualitative methods to balance strengths and weaknesses, combining strengths from one method with the strengths of the other. As both data are gathered simultaneously at the research site, the convergent parallel mixed method design reduces the amount of time needed to collect data compared to sequential approaches (Creswell, 2014).

### 3.3 Data collection tools and techniques

To fulfill the objectives of the study, both primary and secondary data sources were used. The primary source of data was the face-to-face interview with key informants (10), the household survey (265) of the men and women farmers, where participants were selected using a multistage sampling method, and FGDs (4) with farmers. The secondary data sources included the annual reports of PMAMP, AKC, the Agriculture Section Office, MoALD, and related publications. Accordingly, both qualitative and quantitative methods of data collection were used. The qualitative data were obtained from ten KIIs with the public extension officials regarding the delivered paddy seed-related EAS. It was also obtained through the open-ended questions towards the end of the schedule used in the household survey. Four FGDs with men and women farmers were conducted regarding access to EAS. Parallel to this, a quantitative method was used to elicit data and information from the respondents regarding farmers’ perception, attitude to access, and barriers to access to EAS among women paddy seed producing farmers of Chandrapur municipality ward-9.

### 3.4 Data analysis and statistics

After the data collection, the data were subjected to analysis. For quantitative data analysis, the data were tested for normality. The data was subjected to quantitative data analysis. The statistical package for the social sciences (SPSS 27) and Microsoft Excel were used for quantitative data. Socio-economic and farm characteristic of the respondents, like age, gender, education, ethnicity, income, total farm area, land size, farmers’ groups involvement, etc., was described by using simple statistical procedures like percentage, range, frequency, mean, and standard deviation, etc., or inferential statistics, binary logistic regression. Binary logistic regression was applied to find out the degree of relationship between independent and dependent variables for the measurement of gender differences in access to extension services and major farm inputs of paddy seed growers. Qualitative information was collected through focus group discussions, and key informant surveys were processed manually and used in the analysis to complement the quantitative information.

## 4 Results

### 4.1 Distribution of respondents by socioeconomic characteristics

#### 4.1.1 Gender of respondents

**Figure 3:**
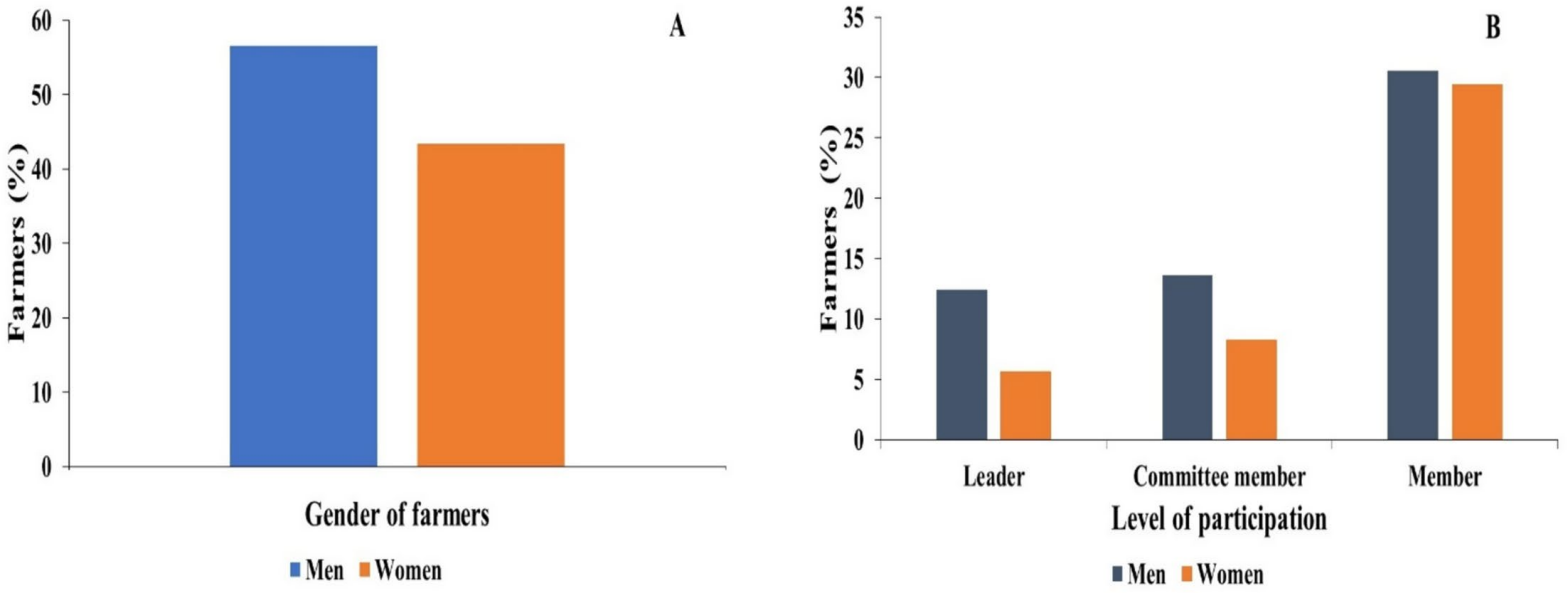
Distribution of respondents based on gender **(A)** and level of participation **(B)** in meetings of respondents.

Among the overall paddy seed farmers interviewed, the majority of household heads were men (56.6%), compared to their female counterparts (43.4%), as shown in Figure 3A. The study showed that the majority of farmers involved in paddy seed production had men as household heads.

#### 4.1.2 Level of participation in meetings of respondents

The bar graph below shows that most of the participation level of respondents is as members (60%), and a few are participants as leaders (18.12%), as illustrated in Figure 3B. Among members, there is no significant difference between men and women; however, in terms of leaders and committee members, a clear distinction is evident between men and women.

#### 4.1.3 Type of farming of respondents

Table 1 presents the data regarding the type of farming. It clarifies that people do not do subsistence farming. They either do commercial or both. Most respondents do commercial farming (61.88%), and a few do both commercial and subsistence farming (38.12%). Among all, men with commercial farming (36.98%) are the most, and women with both farming (18.49%) are the least, keeping subsistence farming aside.

**Table 1:** Distribution of respondents based on education level and the type of farming.

Variables	Description	Men (%)	Women (%)	Total (%)
Education level	Primary	21 (7.92)	38 (14.34)	59 (22.26)
	Secondary	91 (34.34)	48 (18.11)	139 (52.45)
	Higher Secondary	38 (14.34)	29 (10.94)	67 (25.28)
Type of farming	Subsistence	0	0	0
	Commercial	98 (36.98)	66 (24.90)	164 (61.88)
	Mixed	8 (19.63)	93 (18.49)	101(38.12)

#### 4.1.4 Education level of respondents

Table 1 shows the data regarding the education level of respondents. It is clear that most people have studied Secondary level education (52.45%), and few have studied primary level education (22.26%) in total. In particular, we can see that women have attended more years at the primary level than men. Whereas men have attended more compared to women at the secondary and higher secondary levels.

#### 4.1.5 Farming experience and land allocation for paddy seed production

Table 2 shows the mean data for years of production, area of production, and farming experience. The mean years of paddy seed production is 3.72 years with a standard deviation of 2.34. The mean area of paddy seed production is 0.49 years. Similarly, the mean farming experience of farmers is 12.4 years.

**Table 2:** Distribution of respondents based on the farming experience and land allocation.

Variables	Mean	Standard deviation
Years of paddy seed production (Years)	3.72	2.34
Area of paddy seed production (Hectare)	0.49	0.21
Farming experience (Years)	12.4	5.69

#### 4.1.6 Socioeconomic and participatory characteristics of respondents

Regarding ethnicity, the majority of respondents were Brahmin, followed by Chhetri, Madhesi, and Janjati, respectively, as shown in Table 3. The income generated by paddy seed farmers per annum is presented in Table 3, which clearly shows that farmers with annual earnings in the range of 10–15 lakhs account for 43.77%, and those with earnings of 0–5 lakhs account for 18.11%. The age of the respondents was categorized as economically active and inactive. All individuals of either sex who provide the labor supply for the creation of economic products and services are included in the economically active population.

**Table 3:** Distribution of respondents based on the socioeconomic and participatory characteristics.

Variables		Description of variables	Frequency	Percentage
Ethnicity of respondents	Brahmin	Men	63	23.77
		Women	50	18.87
	Chhetri	Men	39	14.72
		Women	21	7.92
	Janajati	Men	4	1.50
		Women	11	4.15
	Madhesi	Men	44	16.60
		Women	33	12.45
Income in lakhs of respondents (Rs.)	0–5	Men	22	8.30
		Women	26	9.81
	5–10	Men	50	18.88
		Women	51	19.24
	10–15	Men	78	29.43
		Women	38	14.34
Age of respondents	Economically active population	Men	146	55.10
		Women	115	43.40
	Economically inactive population	Men	4	1.50
		Women	0	0.00
Frequency of participation in meetings of respondents	Whenever conducted	Men	65	24.53
		Women	21	7.92
	Sometimes	Men	72	27.17
		Women	57	21.51
	Never	Men	13	4.91
		Women	37	13.96

### 4.2 Socio-economic determinants and gender dynamics in access to paddy seed-related extension services and farm inputs

#### 4.2.1 Socio-economic factors influencing gender disparities in paddy seed-related extension services

The logistic regression revealed that while gender had a negative impact on access, female farmers were less likely to use services, income, age, ethnicity, and farm type significantly increased access. These findings are consistent with FGD and KII responses, which indicated that while women faced mobility and time constraints, wealthier, older, male farmers had more contact with extension agents. The qualitative findings that income, social identity, and farm type were more important than household or educational factors were also supported by the fact that larger paddy areas slightly decreased access, while farm ownership, experience, education, family size, and alternative occupation were not significant. 41.6% of the variation in access was explained by the model (Table 4) (Nagelkerke *R*^2^ = 0.416).

**Table 4:** Socio-economic factors influencing gender disparities in paddy seed-related extension services in Rautahat district.

Variables	*B*	S.E.	Exp(B)
Income	0.573	0.225	1.773**
Gender	−0.669	0.334	0.512**
Age	0.149	0.024	1.160***
Ethnicity	0.294	0.130	1.342**
Farm type	1.856	0.403	6.397***
Farm ownership	0.680	0.417	1.974
Farming Experience	−0.055	0.030	0.947
Area of paddy production	−0.066	0.025	0.936**
Education	−0.123	0.234	0.884
Family size	−0.186	0.315	0.830
Alternate occupation	−0.102	0.144	0.903
Intercept	−9.781	2.107	0.000
Log-likelihood	264.725		
Nagelkerke R Square	0.416		

Variable(s) entered: Income, Gender, Age, Ethnicity, Farm type, Farm ownership, Farming Experience, Area of paddy production, Schooling year, Family Size, and Alternate occupation. *** for 1%, ** for 5% and * for 10% level of significance.

#### 4.2.2 Role of gender in access to paddy seed-related extension services

Of the farmers, 56.6% were men and 43.4% were women. Men were more likely than women to have access to extension services (35.1%), but women were slightly more likely to lack access (22.3%), which is shown in Table 5.

**Table 5:** Role of gender in access to paddy seed-related extension services.

Farmers	Access to extension service (%)	Non-access to extension service (%)	Total (%)
Men	35.09	21.5	56.6
Women	21.13	22.26	43.4

The equation of binary logistic regression is:


$$\begin{array}{l} \text{Paddy seed}-\text{related extension services}\\ =-9.781+0.573^{*}\;(\text{Income})-0.669^{*}(\text{Gender})\\ +0.149^{*}(\mathrm{Age})+0.294^{*}(\text{Ethnicity})+1.856^{*}(\text{Farm type})\\ +0.680^{*}(\text{Farm ownership})-0.055^{*}(\text{Farming experience})\\ -0.066^{*}(\text{Area of paddy production})-0.123^{*}(\text{Schooling year})\\ -0.186^{*}(\text{Family size})-0.102^{*}(\text{Alternate Occupation}) \end{array}$$

#### 4.2.3 Socio-economic factors influencing gender disparities in paddy seed-related major farm inputs

Table 6 visualizes the socioeconomic factors influencing gender differences in paddy seed-related major farm inputs. As expected, the influence of gender, farm type, level of participation, and frequency of participation affects farmers’ participation in paddy seed-related extension activities. Likely characteristics of participating farmers were: male; certain farm types; farmers who had committed themselves to the extension, and who participated more frequently. Ethnicity predictably negatively affected participation - some ethnicities were less likely to participate. Other characteristically investigated traits: income; age; farm ownership; farming experience; area of paddy; schooling; other occupation; paddy production years, and were not significantly related to participation. The model likely explains over about 42% of the variation in participation (Nagelkerke *R*^2^ = 0.420).

**Table 6:** Socio-economic factors influencing gender disparities in paddy seed-related major farm inputs.

Variables	*B*	S.E.	Exp(B)
Income	0.198	0.304	1.219
Gender	0.983	0.492	2.674**
Age	−0.040	0.035	0.960
Ethnicity	−0.965	0.317	0.381***
Farm type	1.573	0.571	4.822***
Farm ownership	0.546	0.406	1.726
Farming experience	−0.036	0.040	0.965
Area of paddy production	−0.001	0.034	0.999
Schooling year	−0.412	0.320	0.662
Alternate occupation	−0.205	0.192	0.814
Level of participation	1.741	0.551	5.704***
Frequency of participation	1.148	0.560	3.153**
Years of paddy production	0.109	0.126	1.116
Intercept	−10.334	3.943	0.000
Log-likelihood	154.087		
Nagelkerke *R* square	0.420		

Variable(s) entered on step 1: Income, Gender, Age, Ethnicity, Farm type, Farm ownership, Farming Experience, Area of paddy production, Schooling year, Alternate occupation, Level of participation, Frequency of participation, Years of paddy production. *** for 1%, ** for 5% and * for 10% level of significance.

#### 4.2.4 Role of gender in access to major farm inputs

Of the farmers, women made up 43.4% and men 56.6%. Access to farm inputs was more common among men (20%) than among women (12.5%), and non-access was more common among men (36.6%) than among women (30.9%) which is shown in Table 7.

**Table 7:** Role of gender in access to paddy seed-related extension services.

Farmers	Access to farm inputs (%)	Non-access to farm inputs (%)	Total (%)
Men	20	36.6	56.6
Women	12.45	30.94	43.4

The equation of binary logistic regression is:


$$\begin{array}{l} \text{Access to major farm inputs}=-10.334+0.198^{*}\\ \left(\text{Income})+0.983^{*}\;(\text{Gender})-0.040^{*}(\mathrm{Age}\right)\\ -0.965^{*}(\text{Ethnicity})+1.573(\text{Farm type})+0.546^{*}\\ (\text{Farm ownership})-0.036^{*}\\ (\text{Farming Experience})-0.001^{*}\\ (\text{Area of paddy production})-0.412^{*}\\ (\text{Schooling Year})-0.205^{*}\\ (\text{Alternate occupation})+1.741^{*}\\ (\text{Level}\;\mathrm{pf}\;\text{participation})+1.148^{*}\\ (\text{Frequency of participation})+0.109^{*}\\ (\text{Years of paddy production}). \end{array}$$

## 5 Discussion

This study confirmed that a larger majority of households involved in paddy seed production were led by male members (56.6%), while the remaining households were led by female members (43.4%). This aligns with observations made in Nepal and other developing countries, where there are household headship and decision-making roles in farming that are dominated by males. In these cases, patriarchal norms and inheritance practices often prevent females from being officially recognized as household heads (Gartaula et al., 2012). In the study area, this headship pattern also influenced who primarily interacted with extension agents and input suppliers, with male-headed households more frequently reporting direct contact with service providers Furthermore, differences in education were also noted, with female respondents concentrated at the primary level, and male respondents at secondary and higher educational levels. Studies have reported that a disparity in educational attainment contributes to female participants’ accessibility to agricultural training and extension programs, which can contribute to gender differences in access to new technologies (Ragasa et al., 2013; Abate et al., 2023). Involvement in farming organizations underscored gender disparities. While men and women participated in similar percentages as members of organizations, men were more likely as leaders (18.1%), confirming systematic, entrenched gender barriers to leadership in cooperatives and producer organizations (Curtin et al., 2024; Doss, 2001). Similar findings were reported in Ethiopia and Ghana, where women’s membership often excludes decision-making (Ragasa et al., 2013). Because extension information and subsidized inputs are often channelled through groups and cooperatives, male leadership increased men’s practical access to extension services and major farm inputs. Farming systems were also variable; most respondents practiced commercial farming (61.9%), and subsequently, men farmed commercial only, and women were involved in both commercial and subsistence farming. This also reflects broader evidence that women diversify production strategies for balancing household food security along market participation (Quisumbing et al., 2014).

The average years of paddy seed production (3.72), production area (0.49 ha), and farming experience (12.4 years) indicated that both female and male farmers have a reasonable amount of agricultural knowledge. Studies have indicated that the lack of land, access to credit, and extension services equal to male agricultural producers means that women’s experiential knowledge is not often given due recognition (FAO, 2019; Koirala, 2022). Limited land ownership among women in the study area also reduced their eligibility for certain input support programs and extension-linked schemes. Ethnic origin played a role in some stratification, as most respondents were Brahmins or Chhetris, which corresponds to access to agriculture based on caste designation, and was shown to combine with gender access (Gartaula et al., 2012). Income also revealed a large proportion of men in higher income (annual 10–15 lakhs) than women. Gender disparities in agricultural income are well documented, as barriers to women’s access to productive resources prevent increases in agricultural production and limit access to marketing opportunities (Quisumbing et al., 2014). These findings have important implications, as they indicate that socio-economic factors such as education, leadership, access to land, and income potentially shape some of the gender differences in paddy seed farming. Moreover, they are in line with what other international evidence has shown, that explicitly addressing gender differences through gender-responsive extension services, targeted training, and equitable input access can significantly improve productivity and household welfare (Ragasa et al., 2013). Paddy seed-related extension services are essential for improving agricultural productivity and ensuring long-term sustainability. These services provide farmers with critical information on improved farming techniques, seed varieties, pest management, and other practices that increase yield and environmental sustainability (Thapa, A. et al., 2020). The ability to access and participate in these extension services can vary significantly across different socio-economic and demographic groups. Gender, income, ethnicity, farm type, and other key factors were examined as determinants of access to these services.

With a reasonably strong explanatory power, the logistic regression model used in this study was able to account for about 41.6% of the variation in access to extension services connected to paddy seeds. Higher-income farmers were more likely to use extension services, probably because they could pay for training fees, transportation, and other associated expenses (Ozcatalbas and Ozkan, 2003). Income was found to be a key predictor. Given that female farmers were less likely to receive these services, gender was also identified as a significant factor (Curtin et al., 2024). Some farmers had more access because of their experience and existing connections in the agricultural industry (Galiè et al., 2015). Additionally, it has been demonstrated that access is influenced by ethnicity, with some ethnic groups having somewhat better access than others. This could be due to historical, cultural, or geographical factors that either facilitate or hinder service delivery (Devkota, 2005). Farm type is another significant factor influencing access to extension services. It was discovered that some farm types, especially larger or more specialized ones, were more likely to use extension services. This is probably because these farms have established networks with extension agents and are better equipped to adopt new technology (Galiè et al., 2015). Despite not being statistically significant in this study, farm ownership might have had an indirect impact because owners usually had more autonomy and decision-making authority, which might have improved their access. It’s interesting to note that more seasoned farmers were found to have somewhat lower odds of using extension services, presumably as a result of their greater reliance on traditional knowledge and lack of perception of the necessity for outside assistance. Larger farming areas were also linked to less access, which might have resulted from logistical challenges in getting to vast or scattered plots (Sulaiman, 2010). Lastly, it was discovered that criteria like education, farm size, and participation in alternative occupations did not significantly affect access; this suggests that other factors, namely wealth and unique farm features, were more important.

Access to paddy seed-related extension services is crucial for improving agricultural productivity, resilience, and sustainability. These services provide farmers with valuable knowledge on improved seed varieties, sustainable farming practices, pest management, and technology adoption. It was discovered that socioeconomic and demographic parameters, such as gender, ethnicity, farm features, and degrees of involvement, greatly influenced the ability to participate in extension services. This study’s use of a logistic regression model demonstrated a reasonably significant predictive power, accounting for roughly 42.0% of the variance in participation. Although it was not statistically significant in this study, income was thought to positively affect involvement. Higher-paid farmers were thought to have easier access to resources like equipment and transportation, which would have made it easier for them to take part in extension programs (Ozcatalbas and Ozkan, 2003). With female farmers far more likely to use extension services, gender was found to be a more important determinant (Curtin et al., 2024). According to reports, certain ethnic groups encounter obstacles in obtaining extension services because of social, cultural, or geographic limitations, demonstrating the critical role that ethnicity plays (Devkota, 2005). The likelihood of participation was shown to be higher for some farm types, especially larger, more specialized, or commercially oriented farms. This is probably because these farms have stronger infrastructure, more resources, and a greater ability to adopt new (Galiè et al., 2015). However, neither farm ownership nor farming experience was found to be a significant predictor of participation, indicating that resources and outside assistance might have had a greater impact than ownership status or experience (Galiè et al., 2015). Other factors, like social networks and the availability of extension resources, may have been more important in determining participation rates, as the study found no significant influence from the area of paddy production, education level, or alternative occupations. Nonetheless, it has been demonstrated that regular participation and active engagement significantly raise the likelihood of using extension services. This finding is consistent with the literature, which shows that farmers who are regularly engaged with extension services are more likely to adopt new technologies and improve their productivity (Sulaiman, 2010).

The study’s qualitative findings also highlighted the growing role of women in agricultural decision-making, which may have contributed to their increased use of extension services. Women were seen to play a more active role in farming and agricultural activities as a result of this change in gender roles (Ozcatalbas and Ozkan, 2003). However, it was discovered that marginalized ethnic groups had numerous obstacles, such as a lack of knowledge, difficulties with language, and cultural prejudices. According to Devkota (2005), removing these obstacles will necessitate focused interventions that take into account the particular difficulties these groups experience. According to the study’s farmers, consistent use of extension services was linked to increased adoption of best practices, better knowledge, and higher farm productivity overall. This participation leads to better yields, enhanced skills, and more sustainable farming practices, contributing to improved food security and environmental sustainability.

## 6 Limitations of the study

There are various restrictions on this study. Conclusions regarding the causal relationship between gender, socioeconomic factors, and access to farm inputs and paddy seed extension services are not possible due to the cross-sectional design. The findings are also based on self-reported data, which may be influenced by recall and social desirability bias. Due to financial and resource limitations, the study was also limited to a single district, which limited how broadly the findings could be applied. Despite efforts to maintain objectivity, the researcher’s previous experience working on gender and extension issues in the Rautahat district led to the research being conducted there. This supported field access and contextual understanding, but it may have introduced some interpretation bias.

## 7 Conclusion

The findings from the present study conclude that access to paddy seed-related extension services and farm key inputs is heavily influenced by gender, ethnicity, and farm type, and socio-economic factors such as income, age, and area of production. To ensure inclusive and equitable access to extension services, the study recommends the adoption of gender-sensitive strategies, such as increasing the proportion of female extension agents, offering financial and institutional incentives, developing flexible service models that fit women’s schedules, and promoting joint household responsibilities. Identification and addressing these gender-specific needs are key to creating effective and inclusive extension programs. Ultimately, a gender-responsive strategy will fill the gap for accessing services and allow women and men to improve their farm management and livelihoods. These are measures that are vital in achieving the grand goals of sustainable, inclusive, and equitable agricultural development in plural farming societies.

## Data Availability

The raw data supporting the conclusions of this article will be made available by the authors, without undue reservation.
